# Robotized Fabrication Strategy for Large-Scale 3D Conformal Electronics

**DOI:** 10.3390/ma18215015

**Published:** 2025-11-04

**Authors:** Jiaying Ge, Hao Wu, Hongyang Wang, Dong Ye

**Affiliations:** 1 State Key Laboratory of Intelligent Manufacturing Equipment and Technology, Huazhong University of Science and Technology, Wuhan 430074, China; 13370428095@163.com (J.G.); wanghongyang__1@163.com (H.W.); yedong@hust.edu.cn (D.Y.); 2 Research Center for Advanced Electronics Manufacturing, Huazhong University of Science and Technology, Wuhan 430074, China; 3 Flexible Electronics Research Center, Huazhong University of Science and Technology, Wuhan 430074, China

**Keywords:** conformal electronics, conformal printing, electrohydrodynamic printing, additive manufacturing

## Abstract

Conformal electronics are distinguished by their unique characteristics, such as the integration of structure and function and their conformability with complex geometries. These features unlock a broad spectrum of applications, including structural health monitoring and the creation of metasurfaces. However, the current landscape of large-scale curved electronic fabrication is characterized by a significant gap in specialized equipment and standardized strategies. In this context, we introduce a pioneering strategy that leverages robotized electrohydrodynamic (EHD) printing for the conformal fabrication of large-scale curved electronics on 3D surfaces. This comprehensive multi-robot EHD conformal printing strategy integrates several critical components, including plasma surface treatment, EHD conformal printing, and near-infrared (NIR) sintering processes. These are supported by enabling technologies such as 3D surface reconstruction and precise hybrid positioning. Notably, our strategy achieves 5 µm printing resolution via EHD lithography and 35 µm repeatable positioning accuracy. After plasma treatment, conductive patterns on FR4 substrates reach 5B-level adhesion strength. NIR sintering enables high-efficiency sintering within only 125 s. Seamless integration of these processes into multi-robot collaborative equipment enables the fabrication of large-area conformal electronics, such as 400 mm × 1000 mm unmanned aerial vehicle wings and 650 mm × 350 mm satellite shells, and supports multi-layer systems including wires, LED arrays, antennas, and sensors. This strategy possesses substantial potential to transcend the limitations inherent in traditional fabrication methods, paving the way for new frontiers in conformal electronics across a variety of applications, including smart wings and satellite surfaces.

## 1. Introduction

**Figure 1:**
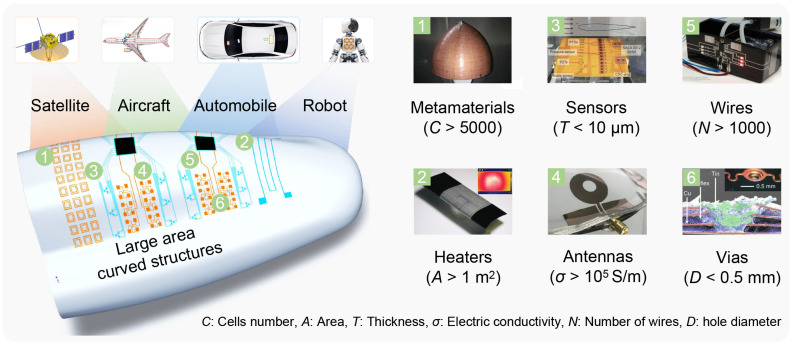
Structural characteristics of large-scale conformal electronics.

Large-scale conformal electronics have garnered significant attention due to their exceptional performance and adaptability. For instance, the “Wing of Tomorrow” project by Airbus integrates embedded sensors to continuously monitor the structural health of large-area curved wing structures. Large-scale conformal electronics are integrated onto the curved structures of satellites [1], aircraft [2], automobiles [3], and robots [4,5,6], as shown in Figure 1a. The electronic devices on these structures encompass metamaterials [7], heaters [8], sensors [9,10], and antennas [11]. They are interconnected by wires [12] and vias [13]. Large-scale conformal electronics pose challenges to the electrical performance and geometric accuracy of manufacturing strategies. For example, electrothermal de-icing heaters on aircraft are larger than 1 m^2^ to cover the entire ice area of the wing [14]. The thickness of sensors on smart skin is less than 10 μm to allow for conformal mounting on curved surfaces [15]. The conductivity of antenna patterns should exceed $1\times 10^{5}\;S/m$ to ensure radiation efficiency. The lead wires used for signal transmission may connect to thousands of sensors distributed throughout the aircraft, indicating a very large scale [2]. Furthermore, the diameters of via holes in multi-layer conformal electronics are less than 500 μm [13]. These fabrication indicators present a significant challenge for the fabrication and integration processes.

Transfer printing has emerged as a leading solution for assembling deformable electronics onto regular curved surfaces [16,17,18,19]. Pfeiffer et al. proposed a transfer printing technology that utilizes air pressure-controlled stamps to slightly deform and directly transfer devices onto bent recipient surfaces [20]. Ko et al. demonstrated the fabrication of a hemispherical electronic eye camera by first creating an island bridge on a planar substrate and then transferring it onto a hemispherical substrate [21]. Additionally, creative structural design can enable the construction of curved electronics to a certain extent. Kwok et al. presented heat-triggered self-folding floral blade structures using origami techniques [22]. Kim et al. developed a process that converts planar materials into 3D structures through magnetic field stimulation, showcasing the principles and characteristics of self-assembly [23]. However, these manufacturing strategies have certain limitations. Firstly, they rely on the use of stretchable materials, which may not be suitable for extreme environments. Secondly, the fabrication area is restricted by the wafer scale and hence cannot be scaled up for a large area.

Direct printing on curved surfaces offers a straightforward approach for achieving structural diversity [24,25]. Various innovative strategies have been developed to improve printing resolution and expand material options. Huang et al. have extensively investigated the fabrication of conformal antennas using piezoelectric inkjet printing technology, and they have enhanced the printing resolution and consistency through the implementation of dimensionality reduction slicing algorithms and droplet injection optimization methods [26,27]. Additionally, aerosol printing has demonstrated the capability to achieve stable jetting within print heights of 2 to 5 mm and print line widths ranging from 10 to 1000 μm [28]. This technique has been employed for printing conformal electronics on 3D surfaces, such as airplane wings or fuselages, characterized by orthogonal planes and acute angles between them [29]. Furthermore, electrohydrodynamic (EHD) printing exhibits multifunctional deposition for dot, line, and film structures through drop-on-demand printing, direct writing, and spraying modes, respectively [30]. Huang et al. have utilized multi-axis conformal EHD printing to successfully manufacture conformal electronics, including antennas, heating grids, and sensors, suitable for application in aircraft smart skin systems [31,32,33]. Especially the EHD lithography technology, which combines EHD printing and lithography, has achieved a conformal printing resolution of 5 μm, making it one of the most effective techniques for fabricating curved electronics on arbitrary surfaces [11].

Whether transfer or direct printing is employed, a comprehensive strategy for the large-scale integration of conformal electronics in engineering applications remains to be developed. The fabrication process necessitates machinery equipped with an expansive workspace, such as industrial robots [34,35]. This paper introduces an integrated approach for the large-scale production of conformal electronics through the application of multi-robot EHD conformal printing. The underlying mechanisms and foundational technologies of conformal EHD printing are thoroughly illustrated, consisting of multi-mode conformal printing techniques, pre-treatment and post-treatment methodologies, and precise positioning and planning strategies. Subsequently, the paper presents a robotized conformal printing system, showcasing its efficacy and adaptability through the successful construction of diverse electronic devices on the surfaces of smart wings and satellite casings. The innovative strategy’s potential is underscored, highlighting its capacity to transform the domain of printed electronics by enabling the fabrication of sophisticated, conformal electronics on a large scale.

## 2. Fabrication Strategy for Large-Scale Conformal Electronics

**Figure 2:**
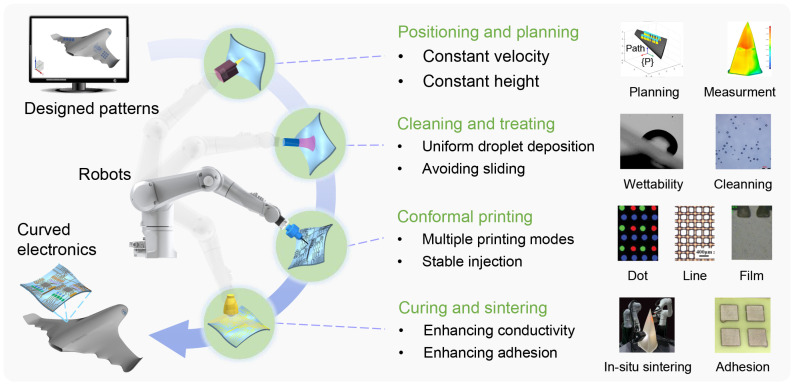
Multi-robot EHD fabrication strategy for large-scale conformal electronics.

Compared to planar printing, conformal printing presents a multitude of unique challenges. The issue of droplet sliding on a curved surface frequently occurs and requires closer attention. Moreover, tasks such as positioning, substrate modeling, and printing speed planning become significantly more complex when dealing with curved surfaces. Consequently, the fabrication of large-scale curved electronics through conformal printing necessitates several critical components, as depicted in Figure 2. These components include precise positioning and trajectory planning to ensure conformal and stable motion, effective cleaning and treatment methods to enable droplet deposition without sliding, in situ sintering processes to enhance film conductivity, and advanced conformal printing techniques for fabricating high-resolution patterns. Here, a multi-robot EHD conformal printing strategy is proposed to deal with these complicated processes.

The high-quality implementation of multi-robot EHD conformal printing relies on four sequential and interconnected core stages, each addressing critical technical requirements to ensure precision, reliability, and performance of the final product. Initially, precise positioning and trajectory planning are essential. Maintaining stability in printing height is crucial for consistent jetting, which depends on an accurate trajectory. On-machine measurement technology is developed to ensure an accurate workpiece coordinate system. Laser and vision-combined positioning technology is employed for global and local positioning, and path compensation is introduced to eliminate motion errors. Subsequently, the substrate must undergo comprehensive cleaning and treatment to regulate surface wettability and eliminate impurities. This process is critical in controlling ink spreading during patterned droplet deposition and ensuring robust adhesion after post-curing. Plasma treatment is used as a method that can change wettability and eliminate impurities simultaneously. Following this, conformal printing entails the precise patterning of curved circuits using a robotized system that adheres to an optimized trajectory. This stage facilitates various conductive and dielectric patterns or films by utilizing multiple printing modes. It is essential in this phase to ensure jet stability by adjusting the printing parameters. EHD printing is adopted for its high resolution and multiple printing modes. Lastly, the metallization or solidification of deposited structures involves in situ sintering. This final process is instrumental in achieving the desired electrical and mechanical properties of as-printed patterns or films. NIR sintering technology is used because it is easy to integrate into the robotized printing systems.

The multi-robot EHD conformal printing strategy delivers a printing resolution of 5 µm, a path error lower than 50 µm (this value is much lower than the absolute motion error of the robot), and a repeatable positioning accuracy of 35 µm. Table 1 compares the proposed multi-robot EHD printing strategy with four mainstream conformal printing strategies, clearly demonstrating its unique advantages in process integration, surface adaptability, and comprehensive performance. Unlike piezoelectric inkjet, extrusion, aerosol, and other EHD-based strategies that only focus on the printing step, our strategy integrates the entire fabrication chain, including plasma surface treatment, conformal printing, and NIR in situ sintering into a single collaborative system. This avoids cumulative errors from disjointed workflows and simplifies operations significantly. In terms of surface adaptability and scale, it breaks the limitations of other technologies such as piezoelectric inkjet and extrusion for low-curvature surfaces and other EHD works for middle-curvature surfaces by enabling stable printing on both high-curvature and large-area substrates such as 400 mm × 1000 mm unmanned aerial vehicle wings and 650 mm × 350 mm satellite shells. Moreover, while maintaining EHD’s inherent ultra-high resolution and broad ink viscosity adaptability, our strategy achieves higher efficiency comparable to piezoelectric inkjet and the lowest cost among all listed technologies, providing a more practical solution for industrializing large-scale conformal electronics fabrication.

## 3. Key Technologies of the Fabrication Strategy

### 3.1. EHD Conformal Printing Technology

EHD printing is a technique where droplets are drawn out and deposited by means of electric field forces [38,39,40]. Thus, it is characterized by high-resolution printing, and it is significantly influenced by the spatial electric field distribution.

#### 3.1.1. Comparison of Three-Axis and Multi-Axis Conformal Printing

**Figure 3:**
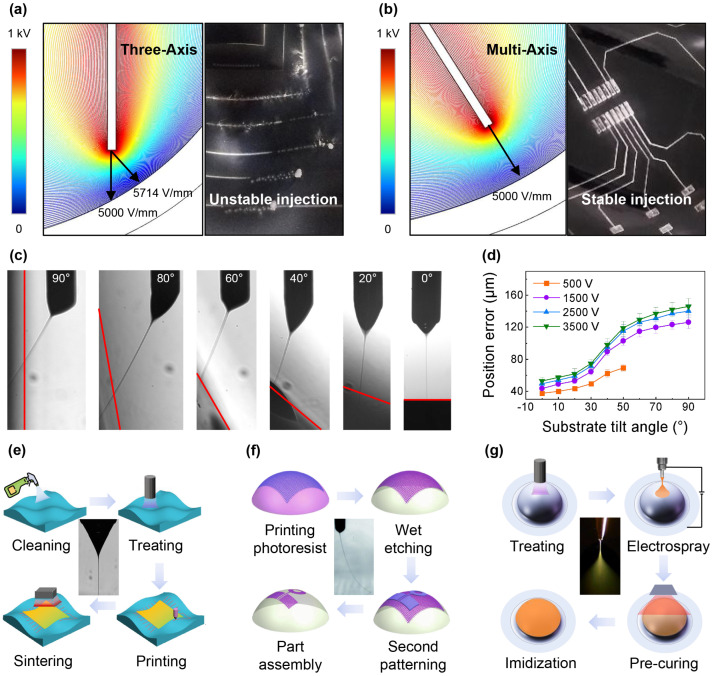
EHD conformal printing technology. Electric field and printed pattern of (**a**) three-axis conformal printing and (**b**) multi-axis conformal printing. (**c**) Droplet deposition behavior under different nozzle tilt angles. (**d**) Comparison of droplet deposition error under different substrate tilt angles and applied voltages. EHD printing process steps of (**e**) Direct-writing, (**f**) EHD lithography, and (**g**) Electrospray.

When printing on nonplanar substrates, the curvature of the surface may change dynamically during the whole printing process. This has a significant impact on the uniformity of the applied electric field, potentially causing instability and failures in the printing process. Therefore, it is essential to manipulate the electric field to mitigate the drawbacks. During EHD printing, the direction with the greatest electric field force is determined by the line from the electrode to the nearest point on the curved surface, which corresponds to the surface’s normal direction (Figure 3a,b). The position and form of droplet deposition are dictated by the magnitude and direction of the strongest electric field force. As for three-axis conformal printing, the deposited position of the droplet deviates from the initially planned trajectory as the discrepancy between the nozzle direction and the electric field direction. As depicted in Figure 3c,d, the droplet deposition position error exhibits an upward trend with increasing substrate tilt angle and voltage. The maximum error recorded in this study was found to be 140 μm. Additionally, the maximum electric field force varies based on the curvature of the surface, resulting in alterations in droplet and volume, thereby introducing instability in the injection process. The utilization of multi-axis conformal printing technology can effectively address such challenges.

#### 3.1.2. EHD Printing Processes Corresponding to Three Printing Modes

By manipulating the process parameters and ink materials, it becomes possible to fabricate micro/nanodots, lines, and films utilizing three distinct jetting modes in EHD printing: drop-on-demand printing, direct writing, and electrospray. These three jetting modes serve as the foundation for the three primary printing processes, including EHD printing, EHD lithography, and electrospray. EHD printing, as an optimal additive manufacturing method, enables the direct deposition of functional ink on a curved surface for electronics manufacturing, leveraging multi-axis motion stages to accommodate various curved geometries seamlessly. Its resolution is 5 µm, which is constrained by the robot motion accuracy. EHD lithography represents a significant advancement in EHD printing technology, providing a pioneering photolithography-based conformal manufacturing process tailored for curved electronics. By deposition of photoresist masks onto a sputtered metal layer, it effectively resolves challenges associated with low printing accuracy and instability on insulating substrates, with 5 µm resolution. Simultaneously, this innovative approach enables the realization of large-area, highly stable production on intricate curved surfaces. Electrospray occurs when an electric field disrupts the liquid cone, resulting in the atomization of the liquid. This phenomenon facilitates the creation of curved dielectric films possessing desirable properties such as insulation, dielectricity, and packing characteristics [30]. Notably, for EHD printing driven by electric fields, the thickness of substrates would influence the electric field strength when thick insulating substrates are applied. For these substrates, alternating voltage is employed, ensuring that the substrate surface uniformly carries charges opposite to those on the nozzle. This ensures a stable potential difference between the nozzle and the substrate regardless of the substrate’s thickness, thus avoiding any impact of substrate thickness on the printing process.

Figure 3e outlines the four steps involved in conformal EHD printing: cleaning, surface treatment, direct writing, and sintering. The process flow for manufacturing conformal antennas using EHD lithography is depicted in Figure 3f. Initially, substrates are cleaned, and a thin metallic film is deposited through magnetron sputtering. The photoresist pattern, generated by EHD direct writing, serves as a mask for the chemical etching process. Subsequently, copper meshes are fabricated using chemical etching techniques. Once the RF components are produced using a distinct EHD lithography method, the remaining electronic parts of the antennas are assembled [41]. Figure 3g illustrates the steps in fabricating polyimide films with the electrospray method. Curved surface substrates are subjected to plasma treatment to adjust surface wettability. The electrospray technique is then applied, followed by pre-curing and an imidization process for the liquid film. After the solidification of the liquid film, the electrospray process can be reiterated to augment the thickness of the deposited films.

### 3.2. In Situ Plasma Pre-Treatment and NIR Sintering Post-Treatment Technology

#### 3.2.1. Plasma Treatment Technology

**Figure 4:**
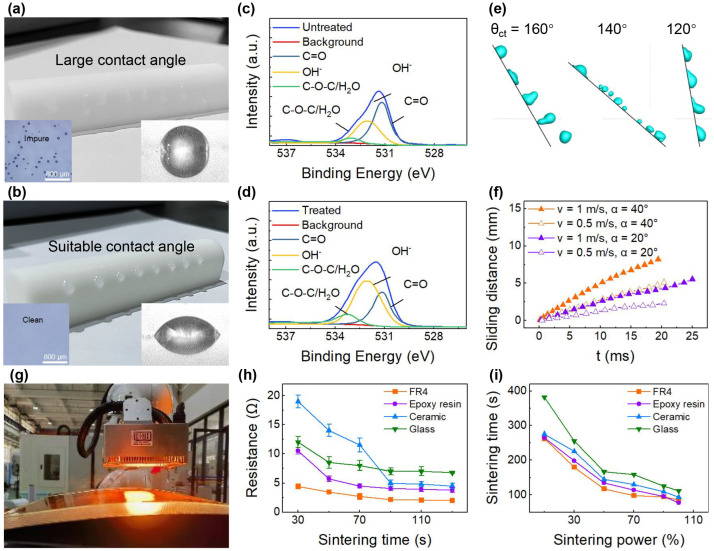
Surface wettability regulation and film sintering processes. Droplet deposition behavior on curved sufaces (**a**) before treatment and (**b**) after treatment. Comparison of surface properties (**c**) before treatment and (**d**) after treatment. (**e**) Droplet sliding behaviors of different contact angles. (**f**) Sliding distance under different impact velocities and the angles of inclination. (**g**) Film sintering by NIR lamp. The (**h**) resistance and (**i**) efficiency of NIR sintering on different substrates.

To improve the surface wettability and remove contaminants of a substrate, plasma treatment has been adopted. This technique renders the substrate suitably hydrophilic, preventing droplets from sliding on its surface (Figure 4a,b). Moreover, fewer impurities remain on the glass substrate after plasma treatment. And the differences in the bonding patterns of polyimide before and after plasma treatment have been investigated by X-ray photoelectron spectroscopy (XPS) (Figure 4c,d). There is a noticeable increase in the presence of hydrophilic groups, such as, particularly, hydroxyl. Additionally, the surface energy rises and results in a decrease in the water contact angle of a surface. These alterations collectively enhance the hydrophilic properties of the polyimide surface, enabling uniform droplet deposition. Moreover, plasma treatment also effectively removes residual charges on the substrate surface, thereby eliminating the interference of substrate surface potential on the spatial electric field distribution during EHD printing. This further ensures the stability of electric field force and improves droplet deposition accuracy.

The plasma jet adopted in this process primarily induces surface chemical modifications, rather than degradation, for common substrates like FR4, polyimide, ceramic, and glass. However, substrates with low thermal stability may face slight thermal damage risks due to the localized high temperature of the plasma jet. To mitigate this, a relatively fast scanning speed (>0.1 m/s) is applied during treatment, which reduces the cumulative thermal exposure of the substrate surface and ensures no observable degradation while maintaining effective surface modification.

Generally, printing on a curved surface would bring some unique ink deposition phenomena that differ from flat substrates, including side-hopping and sliding behavior as droplet spreading (influenced by inertial force) and retraction of droplets (influenced by surface tension) [42]. The deposition of droplets on an inclined surface can manifest in various ways, depending on factors such as the surface wettability of the substrate, impact speed, and the angle of inclination, all of which significantly influence the precise deposition on the target surface [43]. As depicted in Figure 4e, the rebound regime prevails under most impact conditions when the contact angle is set at 140° and 160°. Conversely, the sliding and deposition regimes replace some of the rebound outcomes when the contact angle decreases to 120°. This shift is partly attributed to the larger contact time and area between the droplet and the surface, resulting in increased viscous dissipation. As illustrated in the lower part of Figure 4f, drop sliding distance generally increases with impact velocity and the angle of inclination, as the droplet exhibits a larger tangential velocity [44,45]. However, it is rather challenging to modify and regulate the impact speed and the angle of inclination, for they are determined by the printing process parameters and substrate shape, respectively. To effectively mitigate the droplet sliding, the wettability is regulated.

#### 3.2.2. NIR Sintering Technology

An in situ NIR sintering process has been employed (Figure 4g), which is more effective than heat sintering and easier to integrate into manufacturing equipment. Silver paste straps with 40 mm in length and 800 μm in width on four different substrates: FR4, epoxy resin, ceramic, and glass are fabricated and subjected to sintering. As shown in Figure 4h, the measured wire resistance decreases as the sintering duration increases. And the steady-state resistance achieved at the final state varies across different substrates, with the lowest value obtained on FR4 (2 Ω) and the highest on glass (8 Ω). In addition, the efficiency of NIR sintering in this study refers to the capability of achieving a stable resistance of the conductive patterns under different NIR power intensities. Specifically, a higher efficiency means a shorter time required to reach the stable resistance. Generally, a higher intensity of sintering power would result in a reduction in the required time of NIR sintering (Figure 4i). For example, the sintering process takes more than 250 s at a power level of 10% (0.13 kW), while it is reduced to around 125 s at a power level of 100% (1.3 kW). The time duration varies among different substrates to reach a steady resistance, where the FR4 and glass substrates exhibit the fastest and slowest sintering durations, respectively. Furthermore, after plasma treatment, the adhesion strength of conductive patterns on FR-4 substrates can reach a 5B level.

### 3.3. On Machine Measurement and Hybrid Positioning Technology

Achieving high accuracy in global and local positioning is essential for conformal printing. This is accomplished by integrating multifunctional sensing systems with sophisticated algorithms. The on-machine measurement system provides a foundational coordinate system and detailed geometrical insights into the workpiece, while the hybrid positioning technology, combining laser and vision systems, ensures precise monitoring and control.

#### 3.3.1. On Machine Measurement System

**Figure 5:**
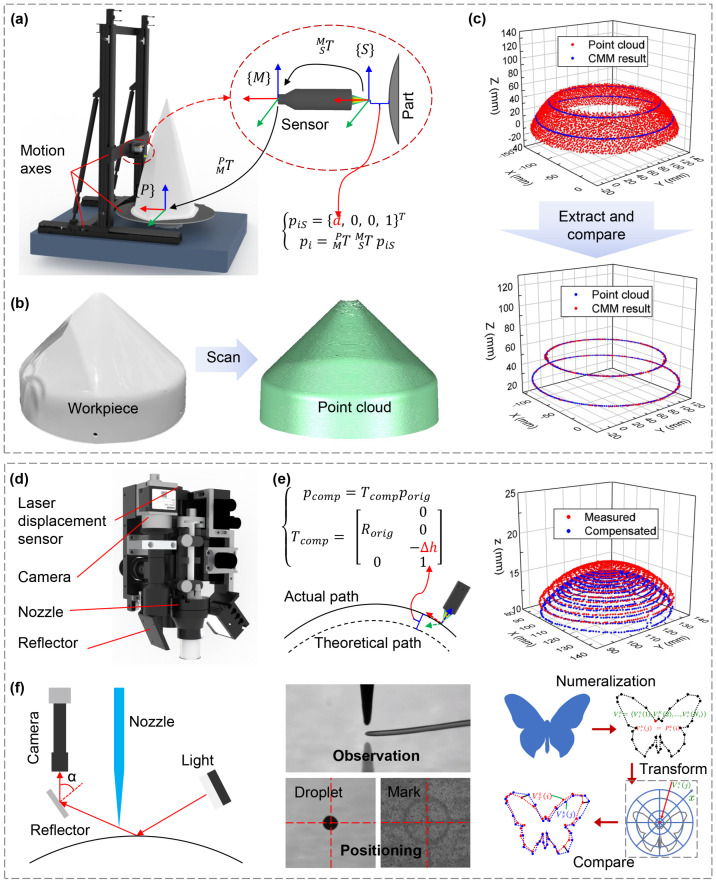
Measurement and positioning technologies. (**a**) On machine measuring system. (**b**) Measured point cloud data. (**c**) measurement accuracy verification (**d**) A home-made integrated smart printhead. (**e**) Principle of print height compensation. (**f**) Principle of vision observation and positioning.

As detailed in Figure 5a, the measuring platform establishes a part frame $\left\{P\right\}$ at the center of a rotary table. A moving frame $\left\{M\right\}$, associated with the laser displacement sensor, is precisely tracked by high-precision optical encoders mounted on the motion axis. The laser displacement sensor measures the gap *d*, which is recorded in the sensor’s focal frame $\left\{M\right\}$. The point cloud data in frame $\left\{P\right\}$ is obtained by transforming the coordinates of $p_{i}$ from frame $\left\{M\right\}$. To validate the precision of the measuring platform, a cone-shaped workpiece was measured and compared with results from a Coordinate Measuring Machine (CMM), as shown in Figure 5b. The diameters of two cross-sections of the workpiece were first measured using a CMM. The platform then captured point cloud data, and circle diameter fitting was performed on the data from two marked cross-sections. The comparison revealed a maximum discrepancy of 17 µm in the fitted diameters between the two methods, confirming the high accuracy of the platform.

#### 3.3.2. Laser and Vision Positioning Technology

The smart printhead, as depicted in Figure 5d, is equipped with a laser displacement sensor for dynamic print height detection and an industrial camera for in situ jetting observation and precise positioning. The laser sensor monitors the print height as the printhead moves along the predefined trajectory, identifying print distance errors Δh. These errors are compensated by adjusting the trajectory normal to the surface, which ensures a consistent average print height and results in a path error lower than 50 µm, as illustrated in Figure 5e. For multilayer interconnection, where precise local positioning is critical, positioning marks are utilized. The relative position between the nozzle and the camera is calibrated by observing the displacement when an ink droplet is deposited and centered in the camera’s field of view as the robot moves. The industrial camera also serves to monitor the jetting status, with an additional reflector mounted for enhanced observation, as shown in Figure 5f. Hand-eye coordination, based on machine vision, enhances the precision of multi-axis systems on 3D curved surfaces. A novel localization model, “surface deformable localization”, is proposed, which relies on shape context and includes an offline and online phase for feature point selection, description, and correlation for accurate localization [46].

## 4. Application of Fabrication Strategies to Equipment and Devices

### 4.1. Multi-Robot Collaborative Conformal Printing Equipment

#### 4.1.1. Conformal Printing Equipment

**Figure 6:**
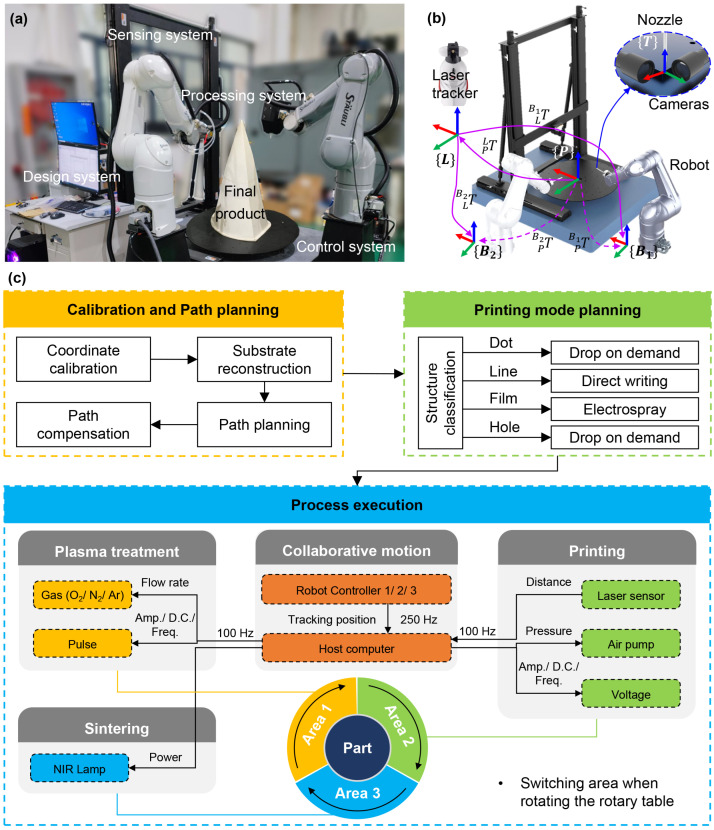
Robotic conformal printing platform (iGreatMaker). (**a**) Photograph of the home-made platform. (**b**) Principle and progress of system calibration. (**c**) Process flow diagram based on the platform.

An advanced robotized printing platform has been designed and developed for printing intricate circuits on large-area surfaces (Figure 6a). This platform consists of a design system, a control system, a processing system, and a sensor system. The control system sends drive commands to the process system to execute the parsed parameterized information. And the processing system carries out the procedure according to the received drive command. Noteworthy features of the manufacturing process system include real-time characterization of the performance status of as-prepared electronics, real-time feedback of monitoring and characterization data to the design system, and dynamic altering of processing parameters and indicators to ensure real-time adjustments in the manufacturing process. The processing system achieves closed-loop regulation and control of the production process through the joint action of the sensing system and the control system, ultimately manufacturing a series of curved electronic products.

To facilitate the fabrication process, a collaborative system comprising multiple robots was employed. These robots can operate within a workspace sphere with a radius of 1050 mm, holding a repeatable positioning accuracy of 35 µm. The platform is equipped with on-machine 3D laser measurement and machine vision modules for global and local positioning, a plasma jet processor for surface treatment, and a UV/photon lamp for post-sintering. The controlling software, programmed in C++, facilitates the connectivity among hardware modules and enables parameter alterations in the manufacturing process. The program also incorporates related algorithms such as “error transfer”, “vision correction”, and “speed planning” to enhance manufacturing accuracy and efficiency.

Figure 6b shows that the home-made robotized manufacturing system employs a uniform part coordinate system $\left\{P\right\}$. The transforming matrix TPBi is used to establish the relationship between the base coordinate system $\left\{B_{i}\right\}$ of the robots and the part coordinate system $\left\{P\right\}$. This matrix is calibrated by adoption of a laser tracker. Specifically, TPBi can be obtained as the product of $T_{L}^{B_{i}}$ and TPL, where $\left\{L\right\}$ represents the laser displacement sensor coordinate system. To further enhance precision, a vision system incorporating two orthogonally arranged cameras is used to calibrate the tool center point. By combining these calibration techniques, the system ensures a remarkable level of accuracy and consistency across the whole fabrication process.

The throughput and the fabrication cycle of the multi-robot collaborative conformal printing equipment are ensured by the partitioned parallel processing of plasma treatment, printing, and sintering, which avoids downtime between processes. Meanwhile, the integrated in situ NIR sintering can complete the solidification of conductive patterns in only 125 s. Additionally, the platform’s control system, programmed in C++, supports seamless compatibility with industrial robot hardware, laying a solid foundation for its potential integration into existing industrial production lines.

#### 4.1.2. Conformal Printing Steps Based on the Equipment

Due to its superior resolution and multi-mode printing capabilities, EHD printing is utilized in conjunction with the multi-axis equipment for direct fabrication of electronics on curved surfaces. To establish a standardized process for fabricating curved electronics, a series of crucial steps (Figure 6c) has been developed. Firstly, a conformal trajectory is generated based on an accurately measured substrate model after the system has been calibrated. This trajectory consists of path points, motion speed/acceleration, and process parameters. To ensure consistent print height, the print trajectory undergoes pre-run and adjustment procedures before initiating the printing process. Subsequently, the manufacturing structure is categorized to determine the appropriate print mode for each circuit layer. Drop-on-demand mode is chosen for point and hole printing, direct writing mode for line patterning, and electrospray for film deposition. Finally, a sequence of processes is executed, including surface treatment, conformal printing, chip mounting, and in situ sintering. The parts are divided into three distinct areas for plasma treatment, printing, and sintering, respectively. During the real-time manufacturing process, the process parameters, including gas flow rate, air pressure, voltage waveform, and the power of the NIR lamp, can be flexibly adjusted along with the robot’s position and printing height. The adoption of multiple robots enables realizing greater efficiency in the manufacturing process, as each robot can focus on its respective task without interruption or downtime.

### 4.2. Conformal Electronics on Smart Skin and Satellite Shell

The robotized conformal printing strategy significantly broadens the manufacturing scale, encompassing diverse structures, elements, components, and devices that ultimately culminate in the fabrication of various products.

#### 4.2.1. Conformal Electronics on Smart Skin

**Figure 7:**
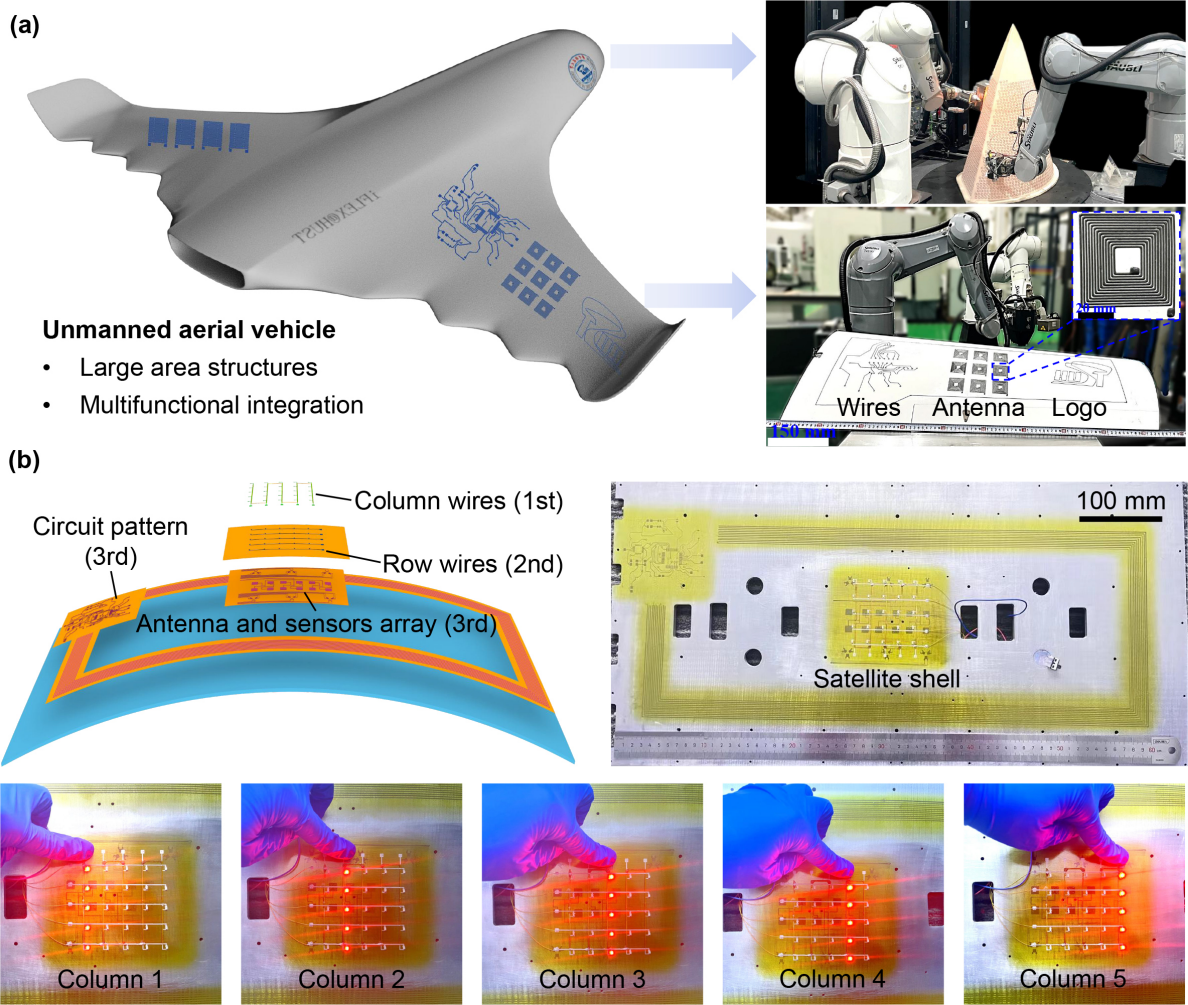
Conformal electronics fabricated by the strategy. (**a**) Integration of conformal electronics onto the wing of an unmanned aerial vehicle. (**b**) Monitoring and LED circuits on a satellite shell.

Notably, one prominent example of large-scale electronics is the smart skin system of aircraft. This sophisticated system incorporates a multitude of functionalities, including interconnect wires for sensor and chip integration, conformal antennas for satellite communications, and electric heating grids for de-icing and ice removal from the wings. To evaluate the efficacy of our proposed methodology, we conducted a series of fabrication experiments on representative circuits within the smart skin system. Figure 7a illustrates the fabricated circuits by using the standard manufacturing process depicted previously. Patterns such as wires, a coil antenna array, and the distinctive “HUST” logo made of solder paste were conformally printed on the wing of an unmanned aerial vehicle (400 mm × 1000 mm), with a printing speed of 6 mm/s and a printing height of 1 mm. It noted that the printed lines exhibit a remarkable uniformity, indicating the successful implementation of the printing height compensation algorithm. And a loop path was precisely printed on the outer ring of the wing, demonstrating the accuracy of the workpiece positioning and the fidelity of the measured-actual models.

#### 4.2.2. Conformal Electronics on Satellite Shell

To further elucidate the fabrication strategy, we have successfully fabricated conformal electronics on a satellite shell with dimensions of 650 mm × 350 mm, as depicted in Figure 7b. The polyimide insulating layers were constructed via the electrospray process, while the conductive layers were produced using the EHD direct writing technique. The resulting three-tier electronic system comprises the following: The first layer consists of column wires, and the second layer comprises row wires, which together form the LED light array. This array allows for individual control over each column. The third layer integrates an antenna and a sensor array, designed for monitoring structural deformation. These experimental outcomes not only corroborate our proposed methodology but also attest to the efficacy and dependability of our in-house developed equipment. They provide empirical validation of the robotized conformal printing technique’s capability to fabricate large-scale electronic systems with precision and reliability.

## 5. Conclusions

This study introduces a robotized conformal printing strategy, specifically designed for fabricating hybrid electronics on 3D curved surfaces. An optimized printing process strategy has been meticulously implemented, encompassing the entire procedure. Digitalized processes are seamlessly integrated through multi-axis printing techniques, which include trajectory planning, precision positioning, and sophisticated robot control mechanisms. Notably, this strategy achieves a remarkable printing resolution of 30 µm and ensures a repeatable positioning accuracy of 35 µm. By integrating various technological advancements, a new realm of versatile conformal manufacturing has been realized. The application of this robotized conformal printing strategy is particularly advantageous for crafting large-area electronic devices on intelligent structures such as smart wings and satellite shells.

Despite the significant potential of multifunctional, large-area, and curved electronic systems, the fabrication strategy involving multi-axis printing systems still faces several challenges. These challenges include the need to enhance accuracy, boost efficiency, and achieve a harmonious multi-process synergy. To surmount these challenges, future research can delve into the application of artificial intelligence methods. Such methods can model the impact of variable inputs like print process parameters and trajectories on print accuracy and quality. This modeling approach will significantly contribute to enhancing the reliability of manufacturing large-area curved surface electronics. Additionally, the exploration of online adaptive trajectory planning algorithms can augment the efficiency of multi-robot collaborative motion. The incorporation of haptic systems into mobile robots can also expand the manufacturing envelope and increase the system’s operational flexibility.

## Figures and Tables

**Table 1:** Comparison of the conformal printing strategies.

Conformal Printing Strategy	Process Integration	Surface Type	Cost	Efficiency	Resolution (µm)	Ink Viscosity (cP)
Piezoelectric inkjet [26,27]	Printing only	Low curvature	Middle	High	>30	10–20
Extrusion [36,37]	Printing only	Low curvature	Low	Low	>70	$10^{2}$–$10^{8}$
Aerosol [28,29]	Printing only	High curvature	High	Middle	10–200	1–2500
EHD (other works) [31,32,33]	Printing only	Middle curvature	Low	Middle	>3	1–10,000
Multi-robots EHD (this work)	Whole process	High curvature, large scale	Lowest	High	5	1–10,000

## Data Availability

The original contributions presented in this study are included in the article. Further inquiries can be directed to the corresponding author.
